# The No-Nonsense Guide to Born-Digital Content

**DOI:** 10.5195/jmla.2019.641

**Published:** 2019-04-01

**Authors:** Paul M. Blobaum

**Affiliations:** University Library, Governors State University, University Park, IL, pblobaum@govst.edu

This publication adds to a growing list of Facet’s No-Nonsense Guides, a series of concise treatments of library and archives topics that are written by experts. Titles in this series give readers an overview and summary of the main points to be considered, along with citations for further reading. Heather Ryan and Walker Sampson address the growing problem of the proverbial gorilla in the room: Librarians are not keeping up with managing the digital materials that are originally created in an electronic format. The print-to-digital transformation has happened so quickly that librarians overall have not kept up with planning for long-term selection, organization, preservation, and access to these materials. While the profession will eventually get to the place that management of “born digital” information will be a core function of archives and libraries, we are not “there” yet.

Ryan and Sampson provide a fine remedy to address this situation. The book is well organized and can be read either as a travel guide, using only the chapters immediately needed, or read cover to cover. Readers should consider reading the “Conclusion” section after the “Introduction,” as the former summarizes what the authors hope they have accomplished, chapter by chapter, and will prepare readers for what is ahead.

While some background and knowledge of archival theory and practices would be helpful in reading this book, it is written for newcomers and nonlibrarians, as well as seasoned professionals. Opaque terminology—such as *Respect des fonds*, an important principle in archival theory where archival records are grouped according to the original way that they were created or received—are explained, and a useful glossary and list of acronyms are provided before the “Introduction.”

Likewise, some knowledge of computer technology would be helpful, but the authors’ main purpose in writing this book is to dispel the myth that a master’s degree in computer science is needed to do this work. The authors make a lot of effort to discuss the basics of computer technology, including encoding, retrieval, and storage, which are critical for successful planning for born-digital materials. This book provides foundational knowledge that can be built into a career of managing digital content.

The book has seven chapters. Chapter 1, “Digital Information Basics,” reviews the basics of computer technology (ones and zeros) and how information is encoded digitally and how it is stored and retrieved in various mediums. Chapter 2, “Selection,” reviews the types of born-digital materials that could be acquired at an institution and discusses policy development. Chapter 3, “Acquisition, Accessioning, and Ingest,” sets forth principles to consider in acquiring content. Chapter 4, “Description,” addresses the collection of metadata and schema. Chapter 5, “Digital Preservation Storage and Strategies,” summarizes preservation practices and planning and budgeting for digital preservation. Chapter 6, “Access,” discusses access policy and legal consideration. Chapter 7, “Designing and Implementing Workflows,” helps readers understand how to do this work in the most productive and cost-effective way.

The potential audience for this book is very broad. This book is an important contribution to librarianship and archive fields, as there has been no comparable monograph published in English about the subject of collecting and providing access to locally created born-digital materials. Librarians who are working with digitization projects of print-to-digital will benefit from this book as well, as the basics of planning for preservation and practical advice on how to back up files and check their fidelity is shared. The discussion of workflows will also be informative.

In addition to the library and archives fields, this book would be helpful to interprofessional work team members, particularly information technology (IT) professionals who are working on these issues at their institutions. Also, archivists and librarians who are advocating for resources to adequately address the need to acquire and preserve born-digital materials that are proliferating across their institutions will want to buy a copy of this book for their deans and directors, and refer to it often in their planning processes. Finally, information managers in organizations that are struggling to handle the growing hoards of files on their servers may find this book useful.

